# Comparative evaluation of Stevia and Xylitol chewing gum on salivary Streptococcus mutans count – A pilot study

**DOI:** 10.4317/jced.55720

**Published:** 2020-06-01

**Authors:** Mitali R. Shinde, Jasmin Winnier

**Affiliations:** 1 Post- graduate, D. Y. Patil School of Dentistry, Nerul, Navi- Mumbai; 2 Associate Professor, D. Y. Patil School of Dentistry, Nerul, Navi-Mumbai

## Abstract

**Background:**

Stevia is a natural sweetener which is used as sugar substitute. It has been suggested that stevia may be anticariogenic. However, there is limited research in this regard. Hence, the present study was designed to assess reduction in *S. mutans* in stevia and xylitol chewing gums. The aim of this study is to evaluate the effectiveness of stevia and xylitol chewing gums on salivary *Streptococcus mutans* count.

**Material and Methods:**

A randomized triple blinded clinical study with a crossover design included twenty healthy children aged 8-13 years with decayed, missing, and filled teeth (dmft)/DMFT index score ≥ 3. Before the test, unstimulated saliva was collected. Children divided into Group I and II were given Stevia and Xylitol chewing gums respectively. Saliva samples were then collected at 15 min (just after spitting) and after 1 h. The amount of *S. mutans* in saliva was evaluated using a selective media (TYCSB). The data were subjected to statistical analysis using statistical software IBM SPSS statistics 20.0 (IBM Corporation, Armonk, NY, USA)

**Results:**

Reduction in *S. mutans* was seen from baseline to 1 hour in both groups in trial and crossover design though intergroup comparison was not statistically significant. There was reduction seen from baseline to 15 minutes and 15 minutes to 1 hour in xylitol and stevia group both in trial and crossover design which was statistically significant.

**Conclusions:**

Stevia containing chewing gum is equally effective to Xylitol chewing gum in reducing salivary *S. mutans* counts.

** Key words:**Stevia, Xylitol , S. mutans.

## Introduction

Chewing gums increase the stimulation of salivary flow rate and is an also effective vehicle for delivering therapeutic agents. Sugar substitutes have been used in chewing gum manufacturing, which can be used after meals and snacks to promote remineralization of enamel (1). The use of chewing gum in dentistry was first studied in 1970s. The Turku Sugar studies were carried out between 1970 and 1973, showed the excellent anti-caries properties of xylitol chewing gum (2).

Stevia, a natural sweetener, has been used in diabetic patients, is a subject of dental research. It has attracted attention as a useful natural substance to treat a variety of aliments due to its anti-bacterial and anti-fungal properties (3). Stevia is derived from Stevia Rebaudiana plant species and composed of stevioside, rebaudioside A, D and E, dulcoside A and B (4). It is 100% natural, zero calories, 200-300 times sweeter than sugar, heat stable, non-fermentable, and has anti-plaque and anti-caries activity (3). Stevia is available in different forms as Table sugar, drops, hard candy, additives in beverages, dairy products, cakes and confectionary but is also added recently in mouth rinse, chewing gum and toothpaste (5). Recent research has demonstrated that plant extracts of stevia may be non-cariogenic in adult population (4).

In vitro study of Stevia extracts against *S. mutans* have revealed that the inhibitory effect shown by alcoholic Stevia extract was superior to the aqueous form, though it was inferior compared to Chlorhexidine (6). Research conducted on the use of 10.6% stevioside mouth rinse has demonstrated significant antiplaque and antigingivitis properties at 6 months trial (7). Stevia was approved to be used as a natural sweetener by FDA in 2008 (8).

Xylitol is synthetically obtained sugar alcohol which is extensively used as a sweetening agent in several commercially available chewing gums (1). There is sufficient evidence supporting the use of xylitol chewing gum to reduce growth and activity of *Streptococcus mutans* and decrease numbers of these microorganisms in both dental plaque and saliva (2,9,10).

Against this background, the aim of our study was to evaluate and compare the effect of two commercially available chewing gums containing Stevia and Xylitol on salivary *S. mutans*.

## Material and Methods

A randomized triple blind crossover clinical trial was conducted to evaluate the effect of stevia and xylitol chewing gum on levels of salivary *Streptococcus mutans*. The minimum required sample size per group was calculated to be 16. As it is a pilot study, a sample size of 10 was selected. Dental examination was performed for 60 subjects in a residential school in Navi-Mumbai, out of which 20 normal healthy subjects between the ages of 8 and 13 years who had DMFT/dmft > 3 were included in study. WHO criteria (1997) was used for assessing caries status (11). The trial protocol was approved by University Research Committee. (Ref.: FRC/2018/Pedo/22). Any subjects with special healthcare needs, systemic diseases, current or recent use of antibiotics and undergoing any dental treatment, orthodontic treatment were excluded from this study. Informed and written consent was obtained from the participating subjects, their parents and school authorities.

-Clinical trial:

At the start of the clinical trial, the subjects were instructed not at eat or drink anything 2 hours prior to the procedure. The study sample was divided randomly into 2 groups i.e. Group 1: Stevia containing chewing gum (Steviadent peppermint flavoured chewing gum) and Group 2: Xylitol containing chewing gum (Trident spearmint flavoured chewing gum). Randomization was done by flipping a coin and 2 colours were denoted to each side of coin. Chewing gum were wrapped in representative colour and were distributed among the subjects. This randomization procedure was carried out by assistant examiner and were decoded at the end of the study. Baseline unstimulated saliva was collected by instructing the subjects to let saliva collect in oral cavity without swallowing for at least 1 minute and then to expectorate in a sterile dispensing cup for 2 minutes (12). One pellet of chewing gum was given to subjects in both groups and they were asked to chew under supervision for a period of 15 minutes. Subjects were asked to chew the gum at their natural chewing frequency for 1 minute followed by 7 minutes on left side and 7 minutes on right side. After 15 minutes, the chewing gum was discarded. The stimulated saliva was collected immediately after discarding the chewing gum by the same procedure and at interval of 1 hour.

-Microbiological Evaluation.

The saliva collecting cups were labelled with subject code and stored at 40 C till all the required samples for each individual was collected, and then transported to the microbiological laboratory within 1 hour in an ice box. The saliva samples were serially diluted using sterile normal saline solution. For dilution, 100 μL of sample was added to 900 μL of sterile saline under aseptic conditions, to prepare 10-1 dilution. Further, 100 μL of 10-1 dilution was mixed with 900 μL of sterile saline. This was repeated until a 10-6 dilution was obtained. The dilutions were then plated on sterile TYCSB (Tryptone Yeast Extract Cysteine Sucrose Bacitracin) agar plate, using a sterile metal spreader. The plates were then incubated at 37ºC for 24 hours. The number of colonies obtained on plates were counted and cfu (colony forming units/ mL) was calculated.

After washout period of 2 days, the same procedure was repeated by interchanging the group i.e. Group 1: Trident chewing gum and Group 2: Steviadent chewing gum.

-Statistical Analysis:

Descriptive and inferential statistical analyses were carried out in the present study. Results on continuous measurements were presented on Mean ± SD. Level of significance was fixed at *p*=0.05 and any value less than or equal to 0.05 was considered to be statistically significant. Based on the results of normality test (Kolmogorov Smirnov & Shapiro Wilk test), it was concluded that part of the data was not following the normal distribution, hence non parametric test were used. Mann Whitney U & Wilcoxon signed Rank test were used to find the significance of study parameters on continuous scale between two groups. Friedman’s test was used to find the significance of study parameters within the group at different time intervals. The Statistical software IBM SPSS statistics 20.0 (IBM Corporation, Armonk, NY, USA) was used for the analyses of the data.

## Results

**Figure 1:**
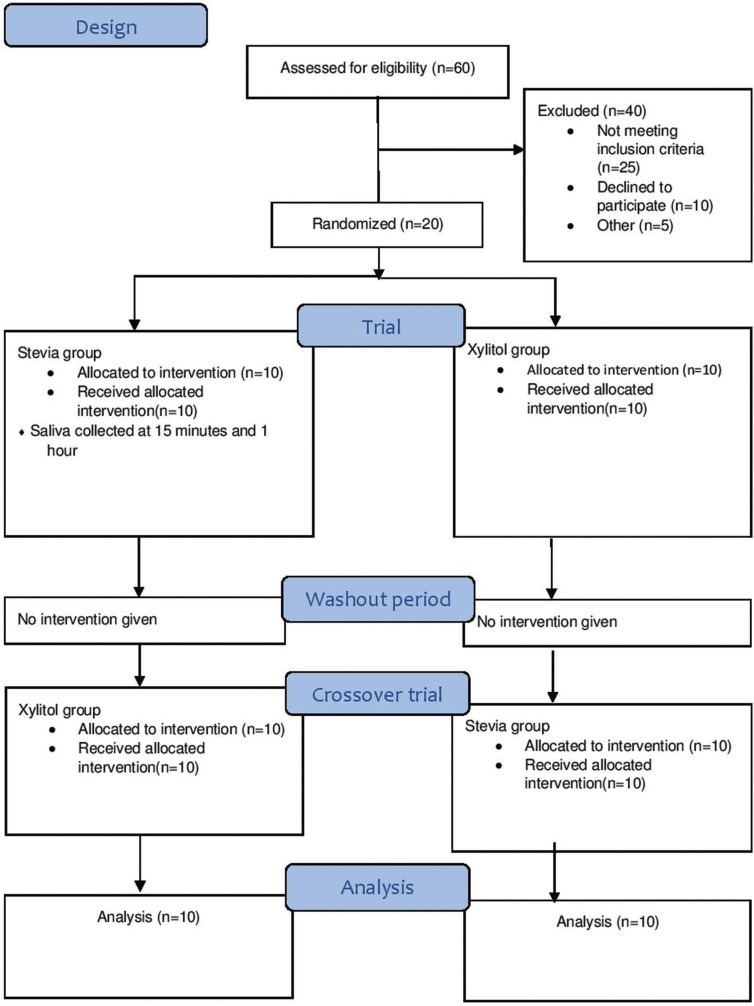
CONSORT 2010 Flow Diagram.

The average age of subjects was 10.25 years. The average DMFT/dmft was 4.34. Table 1 illustrates intragroup comparison for trial and crossover trial. There was reduction in *S. mutans* count from baseline to 1 hour (*P*>0.009) and 15 minutes to 1 hour (*P*>0.047) in stevia group and baseline to 15 minutes (*P*>0.005) and baseline to 1 hour (*P*>0.005) in xylitol group. There was reduction in *S. mutans* count from 15 minutes to 1 hour (*P*>0.047) in stevia group and baseline to 15 minutes (*P*>0.047) in xylitol group in crossover trial group. Table 2 shows intergroup comparison of CFU of *S. mutans* where there was no statistically significant difference seen at baseline,15 minutes and 1 hour both in trial and crossover trial, (Fig. 1).

**Table 1:**
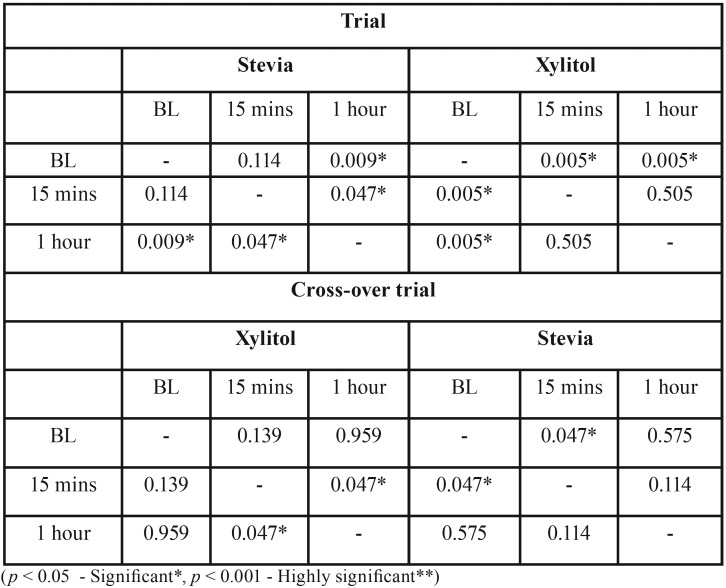
In intragroup comparison, Individual comparison using Friedman’s test and Wilcoxon signed rank test for trial and crossover trial.

**Table 2:**
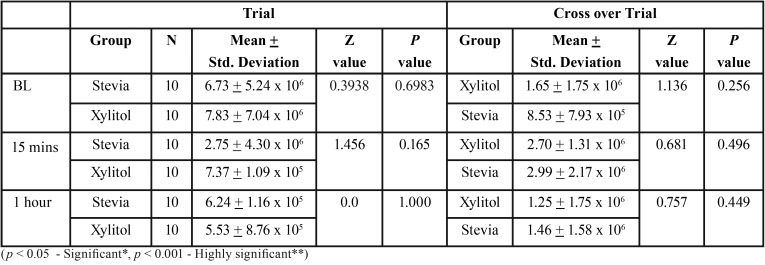
Intergroup comparison of the cfu values in terms of {Mean (SD)} at different time intervals among both the groups using Mann Whitney U test.

## Discussion

Chewing leads to salivary stimulation that neutralizes or elevates the pH, increases buffer capacity and helps in increased removal of bacterial substrates (1). Chewing gums are made from natural or synthetic materials (1). Xylitol is a synthetically obtained sugar alcohol, equivalent in taste to Table sugar and has a half-life of 4 hours (13). It is well established that xylitol chewing gum reduces the growth and metabolism of *S. mutans* (14). Stevia is a naturally derived sugar which is 200 – 300 times sweeter than Table sugar with a half-life of 14 hrs (3,15). It has been recently approved by the FDA as a sugar substitute (8). Previous literature has shown that extracts of stevia is effective agains *S. mutans* and *Lactobacillus acidophillus* (6). However, there is relative paucity of research on the ability of Stevia to decrease oral *S. mutans*. Hence, we attempted to evaluate the effect of Stevia as compared to Xylitol on reduction of salivary *S. mutans* levels.

Children between the age group of 8-13 years were selected for the study, since chewing gum is a well adopted practice among the preadolescent group (16). It was conducted in a residential school for girls thus male subjects could not be selected in the study. Previous research has been established that caries prevalence was not significantly different in boys and girls at an average age (17). Literature has shown a positive co-relation between baseline *S. mutans* count and DMFT/dmft (18-21), therefore in our study subjects with DMFT/dmft > 3 were included. Children with special health care needs, systemic diseases, current or recent use of antibiotics and undergoing any dental treatment, orthodontic treatment were excluded from the study since these conditions may alter the microbial load in the oral cavity (22,23). The frequency of exposure to a cariogenic diet and the form of intake appear to be important factors in the development of dental caries (2). Our study was conducted in a residential school wherein the diet was the similar for all the children for the period of investigation, hence, the type of diet could not possibly modify the factors being examined in the present study. The present study adopted a crossover design so it is more reliable and hence all subjects were both cases and controls. We used commercial preparations of chewing gums as they are readily accessible to general population (9).

At the start of the trial, children were instructed not to eat or drink anything 2 hours prior to procedure, since pH values are lower for 1-2 hours after food consumption. The lower pH increases the bulk and facilitates the attachment of *S. mutans* (1). Saliva was collected by spitting method. Though other methods such as draining, swabbing or suction can be used, we observed that the spitting method could easily be performed by the children. Baseline unstimulated salivary sample was collected. Previous study by Bergstom *et al*. (2016) has revealed no difference in the bacterial profile of the stimulated and unstimulated saliva (24). In our study, TYCSB agar was used to plate the salivary samples for determination of *S. mutans* count. Though MSB agar has been used in several previous studies (25-27), there was no difference in selectivity or sensitivity between TYCSB and MSB in isolating *S. mutans* (28). Literature supporting the use of TYCSB agar state that it results in higher recovery of *S. mutans* than MSB agar (29,30).

There was reduction in *S. mutans* count in both the groups in trial and cross over design though not statistically significant. Similar results were shown by other authors where there was reduction in salivary *S. mutans* count after chewing of xylitol chewing gums (2,31,32). The *S. mutans* count decreased from 15 minutes to 1 hour in stevia group whereas in xylitol group the reduction of *S. mutans* count was seen baseline to 15 minutes. The peak plasma concentration of stevia is observed 15 minutes after a single oral administration (33), which could have led to reduction in *S. mutans* count from 15 minutes to 1 hour. Whereas there is initial fast distribution of xylitol seen in plasma in about 4 minutes (13), which could have led the reduction in *S. mutans* count from baseline to 15 minutes. At baseline to 1 hour, there was reduction in *S. mutans* count in both stevia and xylitol groups which was significant. In vitro studies show reduction in *S. mutans* colonies after use of stevia (5,6), our is the first study to evaluate the effect of Stevia chewing gum on salivary *S. mutans* levels.

## Conclusions

Thus, from results of the present study, it may be concluded that Stevia is equally effective to Xylitol chewing gum in reduction of salivary *S. mutans* levels. However, further long-term studies with larger sample size are needed to compare the efficacy of stevia and xylitol chewing gums in caries-control regimen.
